# Stakeholder Perspectives on Changes in Hypertension Care Under the Patient-Centered Medical Home

**DOI:** 10.5888/pcd13.150383

**Published:** 2016-02-25

**Authors:** Alison J. O’Donnell, Hillary R. Bogner, Katherine Kellom, Michelle Miller-Day, Heather F. de Vries McClintock, Elise M. Kaye, Robert Gabbay, Peter F. Cronholm

**Affiliations:** Author Affiliations: Alison J. O’Donnell, Peter F. Cronholm, Katherine Kellom, Heather F. de Vries McClintock, Elise M. Kaye, Department of Family Medicine and Community Health, University of Pennsylvania, Philadelphia, Pennsylvania; Michelle Miller-Day, Department of Health and Strategic Communication, Chapman University, Orange, California; Robert Gabbay, Joslin Diabetes Center, Harvard Medical School, Boston, Massachusetts.

## Abstract

**Introduction:**

Hypertension is a major modifiable risk factor for cardiovascular and kidney disease, yet the proportion of adults whose hypertension is controlled is low. The patient-centered medical home (PCMH) is a model for care delivery that emphasizes patient-centered and team-based care and focuses on quality and safety. Our goal was to investigate changes in hypertension care under PCMH implementation in a large multipayer PCMH demonstration project that may have led to improvements in hypertension control.

**Methods:**

The PCMH transformation initiative conducted 118 semistructured interviews at 17 primary care practices in southeastern Pennsylvania between January 2011 and January 2012. Clinicians (n = 47), medical assistants (n = 26), office administrators (n = 12), care managers (n = 11), front office staff (n = 7), patient educators (n = 4), nurses (n = 4), social workers (n = 4), and other administrators (n = 3) participated in interviews. Study personnel used thematic analysis to identify themes related to hypertension care.

**Results:**

Clinicians described difficulties in expanding services under PCMH to meet the needs of the growing number of patients with hypertension as well as how perceptions of hypertension control differed from actual performance. Staff and office administrators discussed achieving patient-centered hypertension care through patient education and self-management support with personalized care plans. They indicated that patient report cards were helpful tools. Participants across all groups discussed a team- and systems-based approach to hypertension care.

**Conclusion:**

Practices undergoing PCMH transformation may consider stakeholder perspectives about patient-centered, team-based, and systems-based approaches as they work to optimize hypertension care.

## Introduction

Hypertension is a modifiable risk factor for cardiovascular and kidney disease (1), yet the proportion of adults whose hypertension is controlled is approximately 44% (2,3). Leading primary care organizations introduced the patient-centered medical home (PCMH) to address high costs and poor health outcomes, particularly those related to chronic medical conditions such as hypertension (4). The objective of the PCMH model of care is to have a centralized setting that facilitates partnerships between patients and their personal physicians and, when appropriate, the patient’s family (4).

Several studies demonstrated associations between use of the PCMH model and improvements in the proportion of patients achieving hypertension control (blood pressure <140/90 mm Hg) (5–7). Findings from qualitative studies described electronic health records (EHRs), patient-centered care, the use of protocols or guidelines, and commitment from leadership, providers, and staff (8–11) as facilitators of improving hypertension control. Conversely, participants felt that concerns about the accuracy of blood pressure measurement, a lack of time and resources, a lack of protocols, and patient-level factors (8–10) were barriers to improving hypertension control.

In this study, in contrast to previous work (5–7), we examined hypertension management in the context of the PCMH, a model becoming widely adopted in primary care settings. We also explored perceptions of a range of stakeholders identified by participating sites, including nurses, clinicians, administrators, and social workers. Understanding stakeholders’ perceptions is important for successful hypertension management in the context of the PCMH (11). Our goal was to investigate the changes in hypertension care under PCMH implementation in a large multipayer PCMH demonstration project that may have led to a greater proportion of patients achieving hypertension control. Themes about stakeholder perceptions were used to help understand key factors involved in improving hypertension care in the context of the PCMH.

## Methods

Our study was part of an evaluation of the PCMH transformation process in primary care practices. Practices participated in the first regional rollout of a state-led, statewide, multipayer-supported Chronic Care Initiative. In all, 17 primary care sites in southeastern Pennsylvania participated in a mixed-methods evaluation of the PCMH transformation initiative focused on improving care for patients. Previously published work contains details on the setting and context of this initiative (6). The institutional review boards of both the University of Pennsylvania and Pennsylvania State University approved the study protocol.

Study personnel conducted semistructured individual interviews between January 2011 and January 2012. The research team included individuals with expertise in health services research, qualitative methods, communication, health information technology, primary care, and endocrinology. Research team members developed interview guides based on a literature review of the PCMH and practice transformation. Three standardized interview guides were created for 1) clinicians (physicians and nurse practitioners whose primary role was not care management), 2) staff (medical assistants, nurses, front office staff, care managers, social workers, and health educators), and 3) administrators (office managers and health system administrators/executives). Participants were asked to describe their experiences with implementing the PCMH model at their practice (ie, personal history, their understanding of a PCMH, the process of practice and personal transformation, and lessons learned from the transformation experience). For example, participants were asked “What has the PCMH meant to your practice? What has changed since the start of the medical home/Chronic Care Initiative? How widespread are the changes across the practice?” Participating sites identified key stakeholders to interview. We obtained consent from each participant, and interviews were conducted in private locations at each practice site and lasted approximately 30 minutes. Interviews were conducted until the research team felt that data saturation was reached.

QSR International’s NVivo 9 software was used to organize and manage qualitative data throughout the analytic process (12). De-identified transcripts were entered into an NVivo database. Thematic analysis, which involves iterative development of theories about what is occurring in the data, was used to analyze the data (13–15). Team members coded transcripts independently, making notes of topics emerging from the data. The coding schema was developed through weekly team meetings in which data were explored line by line to reach consensus on topics, address discrepancies, combine similar topics into broader categories, and define the preliminary codes for analysis (16).

## Results

The practices included 7 internal medicine practices, 6 family medicine practices, and 4 nurse-practitioner–led practices in urban (n = 11) and suburban (n = 6) settings. A total of 118 semistructured interviews were conducted with clinicians (n = 47), medical assistants (n = 26), office administrators (n = 12), care managers (n = 11), patient educators (n = 4), front office staff (n = 7), nurses (n = 4), social workers (n = 4), and other administrators (n = 3) (Table).

**Table:** Characteristics of 17 Practices Participating in Study on Care of Hypertension and Transition to a Patient-Centered Medical Home, by Type of Stakeholder Interviewed (N = 118)

Stakeholder	n	Location of Practice, n		Practice Specialty, n			PCMH NCQA Level, a n		
		Urban (n = 11)	Suburban (n = 6)	Internal Medicine (n = 7)	Family Medicine (n = 6)	Nurse Practitioner-Led (n = 4)	Level 1	Level 2	Level 3
Clinicians	47	35	12	21	18	8	15	4	28
Medical assistants	26	20	6	11	8	7	9	3	14
Office administrators	12	8	4	5	5	2	4	1	7
Care managers	11	8	3	3	6	2	2	0	9
Patient educators	4	4	0	1	0	3	3	0	1
Front office staff	7	4	3	4	3	0	0	0	7
Nurses	4	3	1	1	2	1	2	0	2
Social workers	4	4	0	2	0	2	2	0	2
Other administrators	3	3	0	0	0	3	3	0	0

Abbreviations: NCQA, National Committee for Quality Assurance; PCMH, patient-centered medical home.

a NCQA established standards for primary care practices to use in organizing care around patients, working in teams, coordinating and tracking care over time. NCQA accreditation involves categorization into 1 of 3 levels, which represent varying degrees of capability for coordinating care, reporting, and improving quality. Levels range from 1 (lowest level of achievement) to 3 (highest level of achievement).

Participants emphasized the challenges involved in providing hypertension care and discussed the following themes related to changes in hypertension care under the PCMH: 1) patient-centered hypertension care, 2) a team approach to hypertension care, 3) a systems-based approach to quality (Box). Overall, participants were generally optimistic about improving hypertension care and felt that “the collaborative [PCMH model of care] provides a move in the right direction.”

**Box. Central Themes and Subthemes Related to Hypertension Care in the Context of the Patient-Centered Medical Home**

**Table:** 

**Theme**	**Subtheme**
Challenges of hypertension control	Prevalence of hypertension as a health issue Perceptions of hypertension control versus actual performance
Patient-centered care	Patient education related to hypertension Self-management support though personalized hypertension care plans
A team approach to care	Team formation to support hypertension care Expansion of staff roles and responsibilities Staffing and training of personnel providing hypertension care Communication across the health care team
A systems-based approach to quality	Development of infrastructure (ie, electronic health records [EHRs] and protocols) to facilitate hypertension care Creation of patient registries to identify at-risk populations with uncontrolled hypertension Performance feedback to facilitate quality improvement of hypertension care Practice disruption as a result of EHR implementation and registry development Limited customization of the EHR as a barrier to quality improvement

### Challenges of hypertension control

Clinicians described the prevalence of hypertension in their primary care practices and how perceptions of hypertension control differed from actual performance. One clinician described the prevalence of hypertension and the difficulty of expanding services under the PCMH from patients with diabetes to the large number of patients with hypertension. “Moving to other illnesses has actually been a challenge. . . . We have 1,600 diabetics. But we have 9,000 people with hypertension” (Clinician No. 03, Site P).

Clinicians also reflected on the challenges in their practices in achieving hypertension control and the discrepancies between goal attainment and real performance: “I felt terrible about the fact that . . . we can’t control more than 30% or 40% of our hypertension patients. . . . The data was eye-opening” (Clinician No. 01, Site Q).

### Patient-centered hypertension care

A key feature of the PCMH model of care is patient-centered care in which a partnership among practitioners, patients, and patients’ families ensures that decisions respect patients’ wants, needs, and preferences and that patients have the education and support they need to make decisions and participate in their own care (4). Staff and office administrators in particular discussed achieving patient-centered hypertension care through patient education and self-management support with personalized care plans. Practice staff were predominantly responsible for providing patient education. Patient report cards were reported to be useful. “We have nurse case management meetings with [the patients]. We have medical assistants sit with them. They all have their own set of personal goals that they take home with them” (Office Administrator No. 01, Site O). “Well, they come in, have their blood pressure taken, we’ll print out a report card . . . it tells them blood pressure last time and now . . . and it’s teaching them and helping them” (Medical Assistant No. 02, Site N).

### A team approach to hypertension care

Care that is comprehensive and coordinated is central to the PCMH model of care. Under this model, a team of care providers is wholly accountable for a patient’s physical and mental health care needs, including prevention and wellness, acute care, and chronic care (4). In the context of the PCMH, participants across all groups extensively discussed a team approach to hypertension care. For example, role expansion for front desk staff and medical assistants involved more responsibility and engagement with patients. Subthemes included team formation (5 participants), expansion of staff roles and responsibilities (6 participants), staffing and training of personnel (9 participants), and communication across the health care team (7 participants). “Patient care has really improved. . . . Everybody is working more as a team” (Nurse No. 01, Site E).

However, challenges to practice infrastructure change also emerged, including issues related to staffing and training of personnel and communication among the health care team. Clinicians and staff now needed to communicate more, help each other to anticipate problems, and encourage patient involvement in care. This was hampered by high office turnover.

I think the challenge, in addition to the clinicians all getting on the same page, is staff turnover in primary care practices is pretty high. It’s a tough job. It’s hard to find good people, and so when you’re bringing new people in regularly, it’s not like the factory model that it was before. This is sort of a philosophy of care. (Clinician No. 01, Site O)

Staff role identity developed under the PCMH to support coordinated care. Training of personnel and regular meetings within the health care team facilitated this transition. Both clinicians and staff had to realize that care was a partnership between patients and the practices as a whole. “We have meetings once a month with everyone, and we always talk about, okay, this is new now. . . . So everybody hears the same thing. . . . We’re all in the same boat” (Office Administrator No. 01, Site O).

### A systems-based approach to quality

A key feature of the PCMH is a commitment to quality and safety. Clinicians and staff enhance quality improvement to ensure that patients and families make informed decisions about their health (4). Participants across all groups discussed a system-based approach to quality of hypertension care. Subthemes included leveraging the EHR to develop patient registries to identify patients with hypertension (4 participants) and using performance feedback from the EHR to promote quality improvement (5 participants).

One care manager described the EHR as “really state of the art” because of the built-in clinical decision-support system that helped facilitate hypertension treatment. However, EHR customization was limited, making integration of the EHR into practice operations challenging. Although this challenge was partially mitigated by training of staff, several clinicians felt that the EHR system did not aid with the new focus on holistic care and more comprehensive treatment.

There’s a “chief complaint” field where MAs [medical assistants] select from a menu of the chief complaints. And this is an area that does get tricky, and we did a lot of work on training them to pick the appropriate complaints because . . . you’re coming here for diabetes, hypertension, what’s your symptom? (Clinician No. 01, Site X)

Most participants commented that the ability of the EHR to provide feedback to clinicians and staff was invaluable: “I say pull 30 charts of your hypertensive patients out and see how many of them are controlled, and come back and tell me could you do better?” (Clinician No. 01, Site Q).

The data collected from the EHR were used to drive quality improvement projects. For example, as a result of performance feedback, hypertension protocols were revised to ensure the accuracy of blood pressure measurement.

I did a chart review for [physician] and we found that there were discrepancies in the way the measurement was done. . . . We actually changed . . . where the blood pressure cuff was attached to the wall. We had the patients seated versus dangling. We did the blood pressure last, just to allow the best measurement. And then we made an addition in the EMR that not only did the MA [medical assistant] do the blood pressure, but the doctor recorded a blood pressure too. (Care Manager No. 01, Site N)

## Discussion

The PCMH model can improve hypertension control (5–7), and previous data from our study showed a small but significant improvement in hypertension control with implementation of the PCMH model (6). Participants interviewed about their experiences transitioning to the PCMH model emphasized the challenges involved in providing hypertension care and discussed the following themes related to changes in hypertension care under the PCMH: 1) patient-centered hypertension care, 2) a team approach to hypertension care, and 3) a systems-based approach to quality. Stakeholders in practices striving to improve hypertension care in the context of the PCMH transformation process described patient-centered, team-based, and systems-based approaches to care as important and beneficial yet challenging under some circumstances.

Our study has several limitations, which we present before discussing findings. First, stakeholder perceptions provide only a partial view of what occurs in any given PCMH transformation. Several of the stakeholder groups (ie, patient educators, nurses, and social workers) were small compared with others (ie, clinicians and medical assistants). However, the overall sample size was large for a qualitative study. Second, interviewer and analytic team characteristics may influence interviews and analysis. Therefore, the role of the interviewer was clearly explained, and the interview guides were closely followed. In addition, the analytic team had differing backgrounds and disciplinary expertise to reduce bias. Third, the interview guides did not include questions that specifically addressed hypertension. Instead, our analysis focused on people who spontaneously brought up hypertension; we cannot know who may have had experiences related to hypertension care but did not mention their experiences in the interview. We found that hypertension care was a high priority for participating sites. Fourth, cross-sectional data pertaining to a multiyear intervention were collected over a year-long period, which may result in recall bias. Fifth, the sample selected may not have reflected the perspectives of all stakeholders involved in PCMH, namely pharmacists and patients. The selection of study participants by participating sites may be a manifestation of the early stage of PCMH transformation in these practices. Future models may be more likely to encompass a wider range of stakeholders. Finally, the sample came from a single statewide dissemination and implementation model, which may limit generalizability with other models piloted in other regions. However, the sample included a broader range of practice types than previously sampled (17).

In contrast to previous qualitative work (9,10,18), we examined hypertension management in the context of the PCMH, a model becoming widely adopted in primary care settings. Evidence suggests that implementation of key features of the PCMH model of care (patient-centered, team-based, and systems-based care) can improve the management of chronic medical conditions, including hypertension, through reduced health care costs, lower use of health care, and other mechanisms (19,20). In this work, processes of hypertension care were explored from the perceptions of clinicians and administrators, as well as other stakeholders, such as medical assistants, front office staff, patient educators, and social workers, providing a more dynamic and robust examination of hypertension care under the PCMH. Understanding staff needs and alignment of stakeholders’ visions are important components of successful hypertension interventions (11).

Participants expressed excitement about improving hypertension care as part of PCMH transformation despite the process presenting new challenges for practices. In contrast to several other studies that found that cynicism dominated discussion of hypertension care because of a lack of treatment success (8–10), this study found that participants generally were optimistic about hypertension care and hypertension control improving with PCMH implementation. Additionally, although a lack of time and resources and patient-level factors (eg, low socioeconomic status, patient reluctance) have been described as barriers to improving hypertension care (8–10), participants in our study rarely focused on these constraints. In our study, although hypertension care was viewed as challenging, participants emphasized components of care management that were potentially modifiable and identified opportunities for practice growth and development.

Participants across all groups recognized that PCMH transformation required a shift from a focus on acute care to a patient-centered approach focused on managing chronic conditions. Participants, particularly staff and office administrators, discussed patient education and self-management support through personalized care plans as important features of a patient-centered approach to hypertension care under the PCMH. Other research found that patient-centered care is a central component of successful hypertension interventions (8,11,21)

A team-based approach to hypertension care was one of the most frequently discussed themes and appeared to be key to improving hypertension care among participating practices. Participants across all groups recognized that the provision of team-based hypertension care under the PCMH required a team of care providers collaborating to address the health needs of most patients. Training of personnel, especially medical assistants, facilitated role development, and clear communication and regular meetings of the health care team helped to ensure a collaborative approach to care. Evidence indicates that team-based care has increased the proportion of people with controlled blood pressure and reduced both systolic and diastolic blood pressure, especially when pharmacists and nurses were part of the team (22,23). Findings from qualitative research also indicate that better collaboration among health care providers is an important element of successful hypertension interventions (18).

A systems-based approach to quality in hypertension care emerged as the most extensively discussed theme, particularly among clinicians. Achieving a systems-based approach depended on the development of infrastructure, particularly the EHR, to identify at-risk populations with uncontrolled hypertension. Leveraging the EHR to provide performance feedback drove the development of quality improvement initiatives, such as the development of protocols to help standardize hypertension care. However, difficulty with the EHR also emerged as a key barrier to achieving a systems-based approach to quality in hypertension care. Participants noted that the inability to customize the EHR made integration into daily practice operations challenging. Although other studies identified different features of the EHR (ie, alerts, order sets, templates) that contribute to improved hypertension outcomes (24), the ability to customize the EHR is not widely discussed in the literature. EHR systems present challenges that should be addressed to enhance hypertension care under the PCMH.

Hypertension is a prevalent illness and its effective management is challenging. Improving hypertension care under the PCMH may involve a patient-centered and coordinated approach to care delivered by a team of providers who will implement systems-based quality improvements. These data suggest that the training of staff and clinicians may be vital to improving hypertension care under the PCMH. The development of infrastructure, particularly the EHR, may also be vital to improving the quality of hypertension care. Practices undergoing the PCMH transformation may consider perspectives of stakeholders about patient-centered, team-based, and systems-based approaches as they work to optimize hypertension care and improve clinical outcomes.
