# Erratum, Vol. 13, February 25 Release

**DOI:** 10.5888/pcd13.150383e

**Published:** 2016-05-19

In the article “Stakeholder Perspectives on Changes in Hypertension Care Under the Patient-Centered Medical Home,” the order of authors listed in the byline was incorrect. The correct order should have been, “Alison J. O’Donnell, DO, MPH; Hillary R. Bogner, MD, MSCE; Katherine Kellom, BA; Michelle Miller-Day, PhD; Heather F. de Vries McClintock, PhD; Elise M. Kaye, CNM; Robert Gabbay, MD, PhD; Peter F. Cronholm, MD, MSCE, FAAFP.” The change was made to our website on May 3, 2016, and appears online at http://www.cdc.gov/pcd/issues/2016/15_0383.htm. We regret any confusion or inconvenience this error may have caused.

